# Gene Therapy for ALS—A Perspective

**DOI:** 10.3390/ijms20184388

**Published:** 2019-09-06

**Authors:** Marisa Cappella, Chiara Ciotti, Mathilde Cohen-Tannoudji, Maria Grazia Biferi

**Affiliations:** Sorbonne Université, Inserm UMRS 974, Centre of Research in Myology (CRM), Institut de Myologie, GH Pitié Salpêtrière, 75013 Paris, France

**Keywords:** Gene therapy, antisense oligonucleotides, RNA interference, lentiviral vectors, AAV

## Abstract

Amyotrophic lateral sclerosis (ALS) is a fatal motor neuron disease (MND) with no cure. Recent advances in gene therapy open a new perspective to treat this disorder—particularly for the characterized genetic forms. Gene therapy approaches, involving the delivery of antisense oligonucleotides into the central nervous system (CNS) are being tested in clinical trials for patients with mutations in *SOD1* or *C9orf72* genes. Viral vectors can be used to deliver therapeutic sequences to stably transduce motor neurons in the CNS. Vectors derived from adeno-associated virus (AAV), can efficiently target genes and have been tested in several pre-clinical settings with promising outcomes. Recently, the Food and Drug Administration (FDA) approved Zolgensma, an AAV-mediated treatment for another MND—the infant form of spinal muscular atrophy. Given the accelerated progress in gene therapy, it is potentially a promising avenue to develop an efficient and safe cure for ALS.

## 1. Introduction

Motor neuron disorders (MNDs) are a group of neurodegenerative conditions encompassing a wide spectrum of clinical presentations, involving motor neurons (MNs) in the brain and/or spinal cord [1]. Characteristics of MNDs include progressive degeneration of MNs that cause muscle weakness, loss of ambulation, and chronic disability leading to an early death [2]. Among the disorders classified as MNDs, amyotrophic lateral sclerosis (ALS), or Lou Gehrig′s disease, is the most common in adults [2].

ALS is a fatal MND that leads to paralysis and premature death in affected patients. Despite decades of persistent efforts to characterize and develop treatments for this disease no effective cure is available for ALS patients [3]. Clinical care, such as early nutritional intervention with a gastrostomy tube placement, or respiratory intervention with non-invasive positive pressure ventilation, can improve symptoms and prolong the life of patients [4]. Nevertheless, these treatments cannot cure the disease. The failure of traditional pharmacological and interventional approaches can be linked to late diagnosis, complexity of the disease, and challenges involved with the development of a drug that can efficiently reach the central nervous system (CNS) [5].

Gene therapy has emerged as a promising treatment option for ALS patients. Research efforts have been recently translated to ALS patients in which the causative gene and a linked pathological mechanism were identified [6]. Specifically, non-viral gene therapy approaches entered clinical trials for two familial forms of ALS—Cu/Zn superoxide dismutase 1 (*SOD1*)-linked ALS (NCT02623699 [7,8]) and the most common form of ALS caused by mutations in the chromosome 9 open reading frame 72 (*C9orf72*) gene (NCT03626012 [9]). 

Gene therapy can be used to deliver the normal copy of a mutated gene (gene replacement); to reduce the expression of the causative gene targeting its RNA (gene knock-out); to introduce a protective or beneficial factor (gene addition); or to modify the mutant genome (gene editing) [10]. 

To fine-tune gene therapy approaches and maximize chances of a therapeutic effect, several parameters have to be considered. It is important to define the nature of the genetic material (transgene) used for gene correction, the delivery tool used to reach the targeted cells, and the mode of administration, which could be *ex vivo* or *in vivo* [11]. *Ex vivo* gene therapy consists of genetic modification of cells *in vitro* and subsequent implantation into the recipient host [12]. In contrast, *in vivo* gene therapy involves the direct introduction of a therapeutic gene into the target cell using, or not, viral carriers [13]. 

Naked sequences of nucleic acids, i.e., DNA plasmids, RNA- or DNA-based oligonucleotides, or silencing RNA, can be directly delivered to the host cell or they can be chemically modified to enhance their stability [14,15]. A number of genetically-modified viruses are used as gene therapy vectors, which can differ in tropism, host genome interaction, packaging capacity, immune response in target cells, and transduction efficiency [13,16]. 

The aim of this review is to present an overview of gene therapy approaches for ALS. We will describe the journey of these treatments from the preclinical testing to clinical translation, highlighting the potential of viral-mediated strategies to stably transduce MNs in the CNS (Table 1). In particular, we will present the advantages of viral vectors derived from adeno-associated virus (AAV) to stably correct the disease phenotype in the predominantly affected tissues in ALS. We will describe, in particular, how AAV are changing the treatment landscape for familial ALS cases and, thanks to their versatility, could offer therapeutic perspectives for other ALS cases, such as sporadic ALS.

## 2. ALS—A Lethal Disease with No Cure

The majority of ALS cases (90%–95%) are sporadic (sALS) without a family history. However, some cases have a familial history (fALS) of genetic mutations, which represents 5%–10% of all forms [17]. Interestingly, typical mutant genes found in fALS patients are also found in some sALS cases [18,19]. To date, the genetic etiology of approximately two-thirds of fALS and about 10% of the sporadic disease is discovered, identifying over 30 mutated genes [20]. Most of these genes confer a dominant inheritance, which is not always fully penetrant, whereas some of them are recessive or X-linked [21].

ALS is a rare disease but its incidence is predicted to increase over the next few decades due to the progressive rise in the aging population, especially in Western societies [22]. In Europe the incidence is homogeneous (about 2.16 per 100,000 person-years); with more men affected compared to women [23]. Worldwide, it varies between 0.42–5.3 per 100,000 person-years [24]. ALS onset is usually between the ages of 40 and 70, peaking at 58–63 years for sporadic forms and 47–52 years for familial ones [25,26]. Patients are diagnosed based on the presence of an asymmetric, insidious onset of distal painless weakness or muscle wasting as well as a diaphragmatic pattern of respiratory weakness [2]. The main cause of death is respiratory failure, occurring approximately 3–5 years after diagnosis [27]. 

Despite decades of research, only two non-curative drugs are currently available to ALS patients: Rilutek (riluzole) [26] and Radicava (endaravone) [28,29]. While both these drugs can improve the quality of life of patients by slowing down disease progression, they cannot cure the disorder. 

Approved in 1995, Riluzole is a glutamate antagonist that prolongs the survival of patients by a few months [28]. Endaravone, on the other hand, was recently approved (2017) by the FDA. It acts as a free radical scavenger that reduces oxidative stress and protects neurons, slowing disease decline in a sub-population of ALS patients [29,30]. However, the European Medicines Agency (EMA) has not approved this drug, as the Committee for Medicinal Products for Human Use (CHMP) stipulated an additional survival study (https://www.ema.europa.eu/en/medicines/human/withdrawn-applications/radicava).

### 2.1. Familial Cases of ALS, Pathological Mechanisms of SOD1 and C9orf72 Mutations

The most common genetic mutations responsible for fALS are found in *SOD1*, TAR DNA binding protein of 43 kDa (*TDP-43*), fused in sarcoma (*FUS*) and *C9orf72* genes [20]. In about 95% of ALS cases (with or without frontotemporal dementia), the RNA/DNA binding protein encoded by *TDP-43* is a major component of the ubiquitin-positive neuronal inclusion found in post-mortem neural tissues [31,32]. It is, thus, a main pathological hallmark of the disease [31,32]. Mutations in *FUS* are also found in its RNA-binding domain [33,34]. This indicates that abnormal RNA metabolism is an important factor in MN degeneration [35]. Recently, a patient-specific antisense oligonucleotide (ASO) was developed for a young woman with FUS-ALS [36], suggesting the possibility of adapting gene therapy approaches to other fALS cases. However, the development and translation of gene therapy approaches for SOD1-ALS and C9orf72-ALS is more advanced. For these two forms, non-viral mediated approaches are being tested in clinical trials and several AAV-mediated gene therapies were tested in preclinical models of the diseases, which will be translated in the near future. We believe that these efforts will serve as paradigm for the development of broader treatments—for fALS caused by mutations in other genes, as well as for sALS with or without gene mutations. We will, thus, focus on these two forms of fALS, reviewing the progress towards the development of efficient therapeutic approaches. 

**Figure 1:**
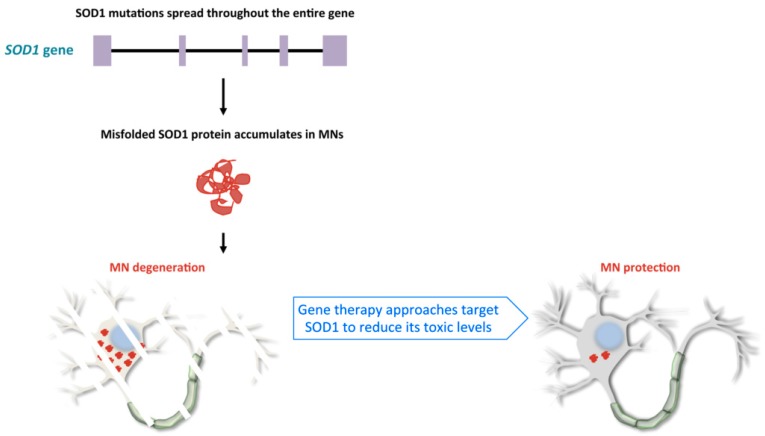
Schematic representation of the human *SOD1* gene, the pathological consequences of its mutations and the effects of the gene targeting approach. The majority of mutations in the *SOD1* gene induce misfolding of the encoded enzyme. Misfolded protein accumulates in protein aggregates and becomes toxic to motor neurons (MN). Gene therapy approaches aimed at reducing SOD1 toxic levels preserve MN degeneration.

The first causative mutations related to ALS were described in the *SOD1* gene in 1993 [37,38]. Since then, more than 180 mutations have been described in this gene (http://alsod.iop.kcl.ac.uk), mainly due to population-based analysis. The *SOD1* gene encodes for the Cu/Zn superoxide dismutase 1 enzyme, an antioxidant homodimeric protein of 32-kDa, whose localization can vary from nucleus to cytosol or mitochondrial intermembrane space. It protects the cell from reactive oxygen species, by converting superoxide into oxygen and hydrogen peroxide [38]. *SOD1* mutations occur in about 12% of patients diagnosed with the familial form, but also in a small percentage of sALS cases [39]. Most of them are point mutations of highly conserved amino acids that can cause conformational instability or misfolding of the protein itself [40] (Figure 1). The mutant protein can trigger neurotoxicity by activating multiple events, such as unfolded protein response, mitochondrial damage, or endoplasmic reticulum stress [40]. The reported heterogeneity in the age of onset and phenotype in patients is due to different types of mutations that result in a gain of toxicity rather than a loss of enzymatic activity [41] (Figure 1). For example, the p.A4V mutation in the *SOD1* gene—the most common variant in North America—is associated with extremely rapid course, usually lasting no more than 12 months, whereas survival may be as long as 80 months for patients with p.E21G, p.G37R, p.D90A, p.G93C and p.I113T mutations. Patients with other mutations can have large intra-familial variations in terms of age of onset and phenotype [27].

**Figure 2:**
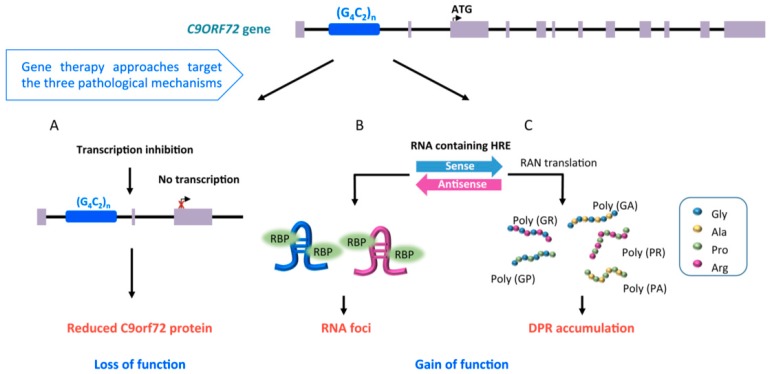
Representation of the pathological mechanisms involved in C9orf72-ALS. The hexanucleotide repeat expansion (HRE) in the *C9orf72* first intron is responsible of C9orf72-ALS through a loss of function or a gain of function mechanism. The HRE can inhibit *C9orf72* transcription causing loss of function (**A**). The expansion can also be bi-directionally transcribed in sense or antisense transcripts that accumulate in RNA foci sequestering RNA binding proteins (RBP) (**B**). The HRE can be translated through a repeat-associated non-AUG-dependent (RAN) translation mechanism producing toxic dipeptides (DPR). Five DPRs have been described: glycine-alanine (GA), glycine-arginine (GR), proline-alanine (PA), proline-arginine (PR), and glycine-proline (GP, generated from both the sense and antisense reading frames) (**C**). The most promising gene therapy approaches will simultaneously address the three pathological mechanisms.

Recent linkage analysis and genome-wide association studies (GWAS) involving people affected by ALS identified a mutant locus on the short arm of chromosome 9 (9p21) that accounts for 40% of fALS and nearly one-quarter of sALS [42,43]. In ALS patients the *C9orf72* gene is characterized by longer GGGGCC (G_4_C_2_) hexanucleotide repeat expansion (HRE) in the first intron (>70 HREs) than in healthy subjects (less than 30 HREs) [43] (Figure 2). This expansion was found in people affected by frontotemporal dementia (FTD), a progressive neuronal atrophy with loss of neurons in frontal and temporal lobes, characterized by behavioral and personality changes, and gradual impairment of language skills. Up to 50% of ALS patients can develop symptoms consistent with the FTD diagnosis with overlapping clinical symptoms and genetic mutations [44].

The *C9orf72* gene is made up of 11 exons and it can be transcribed into three pre-mRNAs: V1, V2, and V3, where transcript variants V1 and V3 retain the HRE in the first intron in ALS cases. Transcript variants V2 and V3 encode the long form of the C9orf72 protein, whereas transcript variant V1 encodes the short form [45]. The C9orf72 protein is highly expressed in neurons and the two isoforms can have different localization in the cells (cytoplasmic for the long protein and nuclear membrane for the short one) [46]. The protein encoded by the *C9orf72* gene might have a role in cellular trafficking due to co-localization with Rab proteins [47,48], regulating endocytosis, and autophagy due to its action as guanine nucleotide exchange factor [49]. 

There are known three non-exclusive mechanisms that are suggested explanations regarding how the HREs cause the disease [44] (Figure 2). First, the presence of repeat expansion causes downregulation of *C9orf72* gene expression leading to a loss of function. Second, HREs are bi-directionally transcribed into RNAs containing G_4_C_2_ and C4G2 repeats that aggregate in nuclei of cells, sequestering RNA-binding proteins (RBPs) into intra-nuclear RNA foci. Third, repeat-containing RNAs can move to the cytoplasm, where they can be translated into dipeptide repeat proteins (DPRs) through a non-canonical translation mechanism known as repeat-associated non-AUG-dependent (RAN) translation [44] (Figure 2). 

The debate concerning these different pathogenic mechanisms is ongoing. It is unclear which one can be considered as the driver of C9orf72-ALS neurotoxicity and, thus, more studies are needed, especially to clarify the function of C9orf72 protein, and how HRE can lead to neuronal death.

### 2.2. Mouse Models for SOD1 and C9orf72-ALS

Several animal models for ALS were generated to understand the pathological mechanisms of ALS and to test therapeutic approaches [50]. Preclinical testing of gene therapies for *SOD1* and *C9orf72*-ALS were mainly conducted in rodent models.

As described before, mutations in *SOD1* gene result in a toxic gain of function mechanisms of the encoded enzyme that for unknown reasons acquires neurotoxic properties. Knockout mice for *SOD1* had a normal development and did not display a MN disease phenotype up to six months of age [51]. In contrast, transgenic mice overexpressing mutant forms of the human *SOD1* gene recapitulate most pathological features of ALS [52]. The most common mouse model for ALS pre-clinical studies was generated in 1994, when multiple copies of the human *SOD1* transgene, carrying a single amino acid substitution of glycine to alanine at codon 93 (SOD1-G93A) was introduced in the mixed B6SJL genetic background [52]. The SOD1-G93A mice had a shortened lifespan with a median survival of about 130 days, progressive paralysis and MN loss in the spinal cord. Similar mouse models with longer lifespans (median survival of about 157 days) were subsequently generated, backcrossing the SOD1-G93A to the *C57BL/6J* (*B6.*SOD1-G93A) [53]. After a spontaneous reduction in the transgene copy number in the *C57BL/6J* congenic mice, an alternative *SOD1* mouse model was generated, with a less aggressive phenotype and a median lifespan of about 300 days (*B6.*SOD1-G93A low copy) (from Jackson Laboratory Bar Harbor, ME USA; Stock No. 002299). A *SOD1*-rat model was also generated by over-expressing the SOD1-G93A transgene, which display paralysis and degeneration of upper and lower MNs and a median survival of about 130 days [54]. These models were used to test gene therapy approaches for SOD1-ALS to support ongoing or planned clinical trials, as detailed in the following paragraphs. 

After the discovery of the pathologic HRE in *C9orf72*, several groups generated and characterized mouse models harboring a bacterial artificial chromosome (BAC) with different lengths of the GGGGCC repetition [9,55,56,57]. In 2015, two groups independently generated BAC mice harboring 500 (C9-500) or from 100 to 1000 repeats (C9-100-1000), respectively [55,56]. These mice only exhibit accumulation of RNA foci and sense DPRs, without the typical ALS neurodegeneration [55,56]. In 2016, two other groups generated transgenic mice expressing 450 (C9-450) and 500 repeats, respectively [9,57]. These mice exhibit RNA foci, DPR accumulations, behavioral, and/or motor alterations. Specifically, the first one, generated by Jiang and colleagues, presented cognitive deficits from 12 months [9]. Interestingly, about 30% of females of the mouse model from Liu *et al.*, developed a rapidly progressive disease with MN loss and decrease in survival (between 20 and 40 weeks of age) [57]. Moreover, when these mice were crossed to knockout mice for *C9orf72*, the motor deficit was exacerbated [58]. Although these different mouse models do not fully recapitulate C9orf72-ALS signs, they were used to test therapeutic approaches for C9orf72-ALS.

## 3. Non-Vector-Based Gene Therapy 

RNA-targeting is a crucial therapeutic strategy for neurodegenerative disorders, like the fALS forms described above, in which RNA/protein accumulation and gain of function mechanism are recognized as the central cause of disease (Figure 1 and Figure 2). 

The main actors in these approaches are ASOs and small interfering RNA (siRNA). The ability of ASOs and siRNA to bind and regulate the expression of mRNA, is a powerful therapeutic strategy for CNS disorders like Alzheimer’s disease, Huntington’s disease, Spinal Muscular Atrophy (SMA), and ALS [15]. Importantly, the first approved gene therapy for SMA is an ASO, called Spinraza. It was designed to correct splicing and induce synthesis of the survival of motor neuron (SMN) protein from the SMN2 transcript [15,59,60,61,62]. Spinraza is currently administered through repeated intrathecal injection to children (including newborns) and adults with SMA [63,64,65,66,67].

### 3.1. Antisense Oligonucleotides in fALS

ASOs are short (13–25 nucleotides), single-stranded nucleic acid, which selectively target and bind mRNA, altering its processing or translation. They can exert a gene targeting effect through different mechanisms. Indeed, ASOs can prevent the interaction with specific RNA binding proteins, involved in RNA splicing or can induce the degradation of mRNA by the activation of ribonuclease H (RNase H) [68]. 

ASOs do not cross the blood–brain barrier; however, when directly delivered into the cerebrospinal fluid (CSF), they are distributed throughout the brain and spinal cord. This has supported their use for the treatment of neurodegenerative diseases [7,15]. 

In 2006, Smith and colleagues demonstrated that direct injections into the CSF via intra-cerebroventricular injections of ASO against SOD1 reduced SOD1 mRNA through RNase H activity, in the brain and spinal cord of SOD1-rats, increasing their mean survival by 10 days [7]. This strategy was tested in patients with SOD1-linked ALS in a clinical trial (NCT01041222). The results of this trial confirmed that intrathecal administration of the ASO in humans was well tolerated with no serious adverse events [8]. The SOD1-ASO, called Tofersen, was then tested in a phase I/II clinical trial, sponsored by Biogen (Biogen Inc., Cambridge, MA USA), (NCT02623699) that showed a reduction in SOD1 protein levels and a trend towards slowing clinical decline in treated SOD1-ALS patients (http://investors.biogen.com/news-releases/news-release-details/biogen-present-new-interim-data-its-phase-12-clinical-study). A phase III trial has been announced, which will test the efficacy and safety of this drug. Importantly, results of pre-clinical testing of the next-generation of ASOs against SOD1 mRNA showed that it was more efficient than the previously tested ASOs—prolonging survival by more than 50 days in SOD1-rats and by almost 40 days in B6.SOD1-G93A mice [69].

Similar to SOD1, different groups developed treatments for C9orf72-linked ALS using ASOs-based approaches. ASOs for C9orf72-ALS were initially tested in induced pluripotent stem cells (iPSC), fibroblasts derived from patients with *C9orf72* mutations and in mouse models [9,48,56,70,71,72]. They were designed to disrupt the hairpin structure of the expansion and prevent RNA binding proteins to be sequestered by G_4_C_2_ repeats [70] or to induce RNase H-mediated RNA degradation [9,56,70,71]. 

ASOs designed to bind within or immediately upstream of the intron 1 (containing the HRE) induce reduction of the RNA foci and DPRs accumulation, preserving the transcript levels, while those binding downstream of the HRE led to significant reduction of the protein [9,48,70,71,72]. Thus, ASOs can be designed to address the three non-exclusive pathological mechanisms responsible for C9orf72-ALS (Figure 2). 

Jiang and colleagues demonstrated that a single, intraventricular administration of a C9orf72-ASO that mediates RNase H degradation could reduce RNA foci and dipeptide aggregates and improve the behavioral and cognitive deficits associated with the C9orf72 repeat expansion in the C9-450 mouse model [9]. Importantly, after the pre-clinical tests in this BAC transgenic mouse model, IONIS (Ionis Pharmaceuticals Inc., Carlsbad, CA USA) in collaboration with Biogen (Biogen Inc., Cambridge, MA USA), has initiated the phase I/II clinical trial (NCT03626012) delivering, via repeated intrathecal injections, the IONIS-C9 (BIIB078) to ALS patients. 

Thus, ASOs delivered to CNS is a feasible treatment for genetic form of ALS. However, repeated direct administrations into the CSF increases the possibility of complications associated with this delivery route. Furthermore, the effect of the ASOs could remain confined to the spinal cord and, given the role of non-neural cells in ALS [73], it may be necessary a widespread delivery of the therapy in CNS and other tissues, such as skeletal muscle. The use of viral vectors and, in particular, of AAV vectors with neural tropism will overcome these issues, enabling persistent and global gene transfer. 

### 3.2. RNA Interference in fALS

RNA interference (RNAi)—a post-transcriptional mechanism of gene silencing mediated by small interfering RNA molecules (siRNA)—is another potential therapeutic approach to target RNA. These are 19- to 23- nucleotide long double-stranded RNA duplexes processed within the cell and assembled into an RNA-induced silencing complex (RISC) to target cellular mRNA. Similar to ASOs, RNAi induce gene expression changes promoting mRNA degradation, alternative splicing manipulation, and transcriptional silencing [74,75]. 

RNAi can be induced by the introduction of synthetic siRNA or by a vector-mediated expression of the precursor short hairpin RNAs (shRNAs), which is subsequently processed in the cytoplasm to siRNA [76]. siRNA can be also expressed by artificial microRNA, in which the shRNA is embedded into sequences of the endogenous short non-coding regulatory RNAs (microRNA) [77]. shRNA or artificial microRNA produce stable effects since they are continuously produced within the cells, unlike the effects of synthetic siRNA that are diluted with cell division or are degraded [76]. 

RNAi for SOD1-ALS was tested to specifically target the mutant SOD1 allele using siRNA *in vitro* and then *in vivo*, using shRNA plasmids delivered in wild-type mice via a hydrodynamic transfection protocol. This approach resulted in limited tissue targeting [78]. 

siRNA directed against the mutant human SOD1 mRNA, was tested in SOD1-ALS mice after local application to the sciatic nerve. With this method the siRNA is retrogradely transported to the perikarya of motor neurons, leading to the inhibition of mutant SOD1 mRNA in B6.SOD1-G93A low copy ALS mice [79]. 

However, difficulties of stably delivering siRNA to specific cell types hampered the translation of these approaches, and the use of viral vector was thus explored for the treatment of fALS. 

The rapid degradation of unmodified nucleic acids by endonucleases necessitates high doses of nucleotides for a therapeutic effect. This may lead to unexpected toxicity or to an increase in off-target effects. Several chemical modifications have, thus, been tested to overcome these hurdles. In particular, different chemistries were developed to protect them from nuclease degradation and/or to achieve targeted delivery at appropriate concentrations [68]. While there are advantages of these modifications, they may lead to complications associated with stability, cellular uptake, and undesirable side effects arising from off-targeting and immuno-stimulation [80,81]. 

Delivery and distribution of ASOs or plasmids encoding for siRNA, can be optimized by conjugating them to various carriers, such as N-acetylgalactosamine, octaguanidine dendrimer, cell-penetrating peptides, or by using liposomes or nanoparticles [81,82,83]. Some of these approaches are currently being investigated under pre-clinical settings and, for example, an ASO conjugated to a cell-penetrating peptide was tested in a severe mouse model of SMA, prolonging survival of treated mice [84]. Future studies optimizing the delivery and bio-distribution of nucleic acids *in vivo* will strengthen the therapeutic potential of these approaches for MNDs. In parallel, viral vectors are being explored as a concrete alternative to efficiently deliver and stably express transgenes, including siRNA and ASOs.

## 4. Viral Vectors for MND Gene Therapy 

The use of viral vectors to deliver therapeutics has completely changed the gene therapy landscape for MNDs. As mentioned above, the blood-brain barrier is a limiting factor for gene delivery into the CNS that prevents entry of therapeutic molecules and virus-mediated gene transfer has been tested to overcome this limitation. Vectors derived from lentivirus and AAV are currently the most frequently used in gene therapy clinical trials for neurodegenerative diseases [85,86]. What makes viral vectors so attractive for MNDs and many neurological conditions? It is their high efficiency for neuron transduction [87]. Among viral vectors, AAV are frequently used in clinical trials for rare disease (i.e., for the treatment of hemophilia A and B—NCT03588299, NCT03001830, NCT02396342, NCT01620801, and NCT03569891), neurological disorders (i.e., Parkinson’s disease—NCT01621581, NCT01973543, NCT03562494, NCT03065192, NCT02418598, and Alzheimer’s disease—NCT00087789, NCT03634007) and are concrete treatment perspectives for patients suffering from MNDs.

### 4.1. Gene Therapy Mediated by LV

Lentiviral vectors (LV) derive from Lentivirus, a member of the *Retroviridae* family. They distinguish in two groups: the primate-like, based on human immunodeficiency virus (HIV) and non-primate vectors, such as the ones derived from equine infectious anemia virus (EIAV) [85]. 

There are different generations of LV according to the packaging plasmid used for their production [88]. The last generation of LV (third-generation), has a simplified genome organized in *gag*, *pol*, and *env* genes, encoding for the structural proteins of the viral capsid, the proteins responsible for viral DNA synthesis, and the viral envelope, respectively. In addition, the viral genome contains two long terminal repeats (LTR), with elements required for gene expression, reverse transcription and integration into host chromosome [88]. To make these vectors useful and efficient for gene therapy and harmless for humans, many accessory and regulatory genes, like *tat*, responsible of oncogenesis, or *vpr*, involved in apoptosis, have been eliminated.

LV was considered the vector of choice for gene therapy due to its ability to transduce dividing and non-dividing cells (such as neurons), its ability to naturally penetrate an intact nuclear membrane [89] and its large cloning capacity (8–10 Kb) [85,90]. LV can induce a stable long-term expression through integration into chromosomes of host cells, in the absence of inflammation [91]. 

There are different pseudotypes of LV with different envelopes, responsible for viral tropism. The most frequently used is the vesicular stomatitis virus glycoprotein (VSV-G), which has a broad tropism *in vitro* and a preference for neuronal tropism *in vivo* [85]. However, while this vector has a high transduction efficiency and stability [92], its unselective tropism and difficulty to enter the CNS without invasive delivery methods, tempers its use as a gene therapy vector [92,93]. Interestingly, LV pseudotyped with the rabies virus envelope glycoprotein (RVG) transduces motor neurons *in vivo* through retrograde transportation—an interesting property to test treatments for MNDs. 

By combining different domains of VSV-G and RVG, LVs with highly-efficient retrograde (HiRet) or neuron-specific retrograde (NeuRet) gene transfer properties were generated [94]. The LV-HiRet, pseudotyped with fusion glycoprotein B type 2, can efficiently transduce motor neurons in the lumbar spinal cord when injected into wild-type mice muscles [95]. However, the intramuscular delivery of viral vectors could be likely impaired by neuromuscular denervation and defective axonal transport, typical signs observed in ALS [73,96]. The translation of these vectors for the treatment of ALS patients, thus, remains questionable. 

LVs were used to test treatment for fALS, using the RNAi approach. Raoul and colleagues demonstrated that bilateral intraspinal injection of a VSV-G LV inducing RNAi-mediated silencing of SOD1 in 40-days-old SOD1-G93A mice, reduced disease onset by 20% and prolonged disease progression, protecting MN loss [97]. Independently, Ralph and colleagues, demonstrated that intramuscular injections of a RVG-LV encoding a shRNA to SOD1 induced a delay in the onset of ALS symptoms, by more than 100% and increased survival by 77% [98] in SOD1-G93A mice injected at seven days of age. These studies were not translated to the clinics, but set the ground for AAV-mediated silencing strategies for SOD1-ALS.

Although LVs have many features that make them suitable for the gene therapy, they have drawbacks that limit their use for *in vivo* gene transfer. These include limited transduced area, around to 500–700 µm from the site of administration [99], their large size (100 nm in diameter), low viral titres and broad tropism. Moreover, their property to integrate genome into host cellular chromatin is a risk for mutagenesis. However, genome integration makes these vectors a favorable choice for *ex vivo* gene therapy, as shown by Suzuki and Svendsen, who demonstrated the therapeutic benefits of *ex vivo* gene therapy in SOD1 rats [100]. This approach is currently being investigated in a clinical trial for ALS at Cedars-Sinai Medical Center, United States, in which mesenchymal stem cells are LV-corrected with glial cell line derived neurotrophic factor (GNDF) transgene and are then infused into the patient’s spinal cord. 

### 4.2. Gene Therapy Mediated by AAV 

Viral vectors derived from AAV virus are widely used for *in vivo* gene transfer in neurodegenerative disorders. AAV is a non-enveloped, single stranded DNA-containing virus, belonging to the *Parvoviridae* family [101]. The viral genome is of around 4.7 Kb and comprises two open reading frames, *rep* and *cap* genes, flanked by inverted terminal repeats (ITRs) on the both ends. The *rep* gene encodes four proteins involved in AAV replication, transcription, integration, and encapsidation. The cap gene produces three structural proteins (VP1, VP2, and VP3), which interact to form a capsid of an icosahedral symmetry. The *cap* gene encodes an additional, non-structural protein called the Assembly-Activating Protein (AAP), which is essential for the capsid-assembly process [101]. To produce recombinant AAV vectors, all of the viral sequences are eliminated except the ITRs that function as packaging signals and by priming second-strand synthesis in host cells. This represents an important safety advantage [102,103,104]. 

AAVs transduce both dividing and non-dividing cells and mediate long-lasting transgene expression without adverse effects. AAVs also exhibit important advantages compared to LV, including a specific tissue tropism of the different serotypes, a higher safety profile and transgene expression levels, a larger vector spread and the persistence of its genome predominantly as extra-chromosomal episomes, thus reducing the possibility of insertional mutagenesis [16].

Hundreds of AAV serotypes have been described and many target specific tissues [105]. The different serotypes are defined by the protein amino acid structure of the capsid that are responsible for the tissue tropism, distribution, as well as the susceptibility to circulating antibodies [106]. Among these serotypes the ones principally used in pre-clinical tests for ALS for their ability to target MNs, are serotype 9 [107,108] and rh10 [109,110,111]—see below. 

AAV-mediated gene expression can selectively target neurons or glial cells with the use of neuronal- or glial-specific promoters, as previously described for LV and adenoviral vectors [112,113]. Axonal transport was initially exploited for vector spreading within the CNS, in a retrograde and/or anterograde direction. Viral vectors can cross synaptic connections and transduce neurons in different regions [106]. AAV serotype 9, for instance, undergoes both anterograde and retrograde transport, which contributes to its wide distribution throughout the CNS [114]. AAV vectors packaging a double-stranded genome have also an increased transduction efficacy [115]. These are called self-complementary (sc) AAV vectors that provides substantial advantages among standard single-stranded (ss) genomes for rapid and efficient transduction in many organs, including the brain, even though it reduces transgene capacity to about 2.5 Kb [107]. 

A breakthrough in the treatment of MNDs emerged with the finding that sc viral vectors, derived from AAV9 cross the blood brain barrier in animal models [107,116]. This method efficiently transduces the CNS, including spinal MNs, following systemic delivery. Using this approach it was demonstrated that the intravenous (IV) delivery of an AAV9 vector encoding the Survival of Motor Neuron cDNA rescues a severe animal model of SMA [108,117,118]. Remarkably, this therapeutic strategy showed promising outcomes in SMA patients [119] and recently received marketing authorization from the FDA for the treatment of pediatric patients (https://www.fda.gov/news-events/press-announcements/fda-approves-innovative-gene-therapy-treat-pediatric-patients-spinal-muscular-atrophy-rare-disease). The AAV9-SMN approach is currently being tested in clinical trials for older forms of SMA (NCT03381729), (https://www.avexis.com/research-and-development), opening perspectives for the application of similar methods in ALS [6].

Recently, AAV9 vectors have been used to deliver shRNA to SOD1 in SOD1-G93A mice after IV injection at birth, which prolonged the median lifespan of treated mice by 39% [120]. This approach reduced the synthesis of mutant SOD1 delaying disease onset and slowing disease progression and is currently under pre-clinical development (AVXS-301-AveXis, Bannockburn, IL USA) [121]. Other groups used an AAV serotype rh10, which also efficiently transduces the CNS [109], to deliver artificial microRNA (miR) into SOD1 mice. Wang and colleagues intrathecally injected an AAVrh10-miR in B6.SOD1-G93A mice at about 60 days of age and prolonged their mean survival by 11% [122]. In parallel, Borel and colleagues tested a similar vector, carrying two copies of the miR sequence to SOD1, by intravenous injection in SOD1-G93A mice. Mice were treated at about 60 days of age and their median lifespan was prolonged by 21% [123,124]. An AAV9 was also used to deliver two miR sequences to SOD1 in newborn SOD1-G931 mice, using intra-cerebroventricular delivery, which prolonged median survival by 50%. Importantly, the injection of an AAVrh10-miR to SOD1 was proven effective in cynomolgus macaques without showing adverse effects [125]. Moreover, based on these studies, AAVrh10-mediated approaches to silence SOD1 are currently under pre-clinical development at Voyager Therapeutics Inc, Cambridge, MA USA (VY-SOD101) and at Apic Bio Inc., Cambridge, MA USA (APB-102).

AAVrh10 was also used to deliver AS sequences to silence SOD1 expression in SOD1-G93A mice. The silencing was based on the activation of the Nonsense Mediated Decay (NMD) pathway, an mRNA surveillance mechanism that detects and degrades abnormal transcripts [126]. The AS sequences were designed to induce the skipping of exon 2 of human SOD1 pre-mRNA in order to produce an mRNA with a premature stop codon activating RNA degradation [127]. The AS sequences were delivered using a U7 small nuclear RNA [128] and were injected through combined IV and intracerebroventricular delivery of the AAVrh10 particles. This approach mediated survival increase in SOD1-G93A mice injected either at birth or at 50 days of age (by 92% and 58%, respectively). The AAV-U7-AS strategy combines the properties of ASO and AAV vectors and is a viable alternative to viral-mediated delivery of siRNA [127]. 

Finally, AAV-mediated expression of silencing sequences was also tested in C9orf72-ALS. Peters and colleagues tested AAV9-mediated silencing of the C9orf72 transcript in cultures of primary cortical neurons from C9-500 BAC transgenic mice, using artificial miR sequences. Using this approach, they could attenuate expression of the C9orf72 BAC transgene and the poly (GP) dipeptides [55]. Recently, the Biotech uniQure (uniQure Inc., Lexington, MA USA) demonstrated that artificial miR can reduce sense RNA foci and repeat-containing C9orf72 transcripts after intrastriatal injection in C9-100-1000 mice at about 90 days of age. They used an AAV5 vector targeting only certain brain regions, like frontal cortex and midbrain area [129,130].

The results of pre-clinical tests of AAV-mediated gene therapy approaches for SOD1- or C9orf72-ALS open concrete perspective for the translation of these approaches to ALS patients.

## 5. Genome Editing for fALS

Genome editing approaches have been recently tested *in vitro* and *in vivo*, for different genetic diseases [131], including SOD1 and C9orf72-ALS. The most current being the use of RNA-guided Cas9 endonuclease from clustered regularly interspaced short palindromic repeats (CRISPR)-associated Cas system that precisely corrects the genetic defects [132,133,134]. Using AAV vectors expressing a short Cas9, derived from *Staphylococcus aureus*, and single-guide RNA directed to SOD1 exon 2, SOD1 expression was disrupted in SOD1-G93A mice [135]. This approach prolonged mice survival by 30 days, however it induced a seven- or 14-fold increase of indels in the human *SOD1* gene in thoracic or lumbar MNs, respectively. The effects of such genomic alteration in humans need to be carefully evaluated.

Recently, a CRISPR-Cas9 plasmid was nucleofected into iPSCs from C9-ALS patients and deleted the repeat expansion, offering a proof-of-concept for genomic correction in this disease [136]. 

Although genome editing is the Holy Grail to achieve definitive correction of a genetic mutation, some aspects, such as target specificity and immunogenicity, need to be managed to ensure safe translation to humans. 

## 6. Delivery of Neurotrophic Factors as Potential Treatment for All ALS Cases

The major parts of ALS cases are sporadic and the precise pathological mechanism is still not understood. One of the strategies developed to target mechanisms common to both fALS and sALS is to raise neuroprotection through trophic factors. 

Neurotrophic factors have an important role in development, plasticity, neurogenesis, disease, and injury of the nervous system. Therefore, brain-derived neurotrophic factor (BDNF), glial cell line derived neurotrophic factor (GDNF), insulin-like growth factor 1 (IGF-1), and vascular endothelial growth factor (VEGF) have been used to treat ALS [137]. Viral gene transfer can deliver and lead to transgene expression of these neurotrophic factors, increasing their bioavailability. Wang and colleagues demonstrated that AAV2-mediated expression of GDNF following intra-muscular injection, delayed disease onset and prolonged survival in SOD1-G93A mice [138]. Similarly, in 2003, Kaspar et al. demonstrated that the delivery of IGF-1 mediated by AAV and VSV-G LV increased survival of SOD1-G93A mice [139]. In 2004, Azzouz et al. reported that a single injection of a VEGF-expressing RVG-LV into various muscles delayed onset and slowed progression of ALS in SOD1-G93A mice, even when the treatment was initiated at the disease onset [140]. Two other studies utilized an AAV2-based vector to administer IGF-1 intraparenchymally in SOD1-G93A mice [141] and rat [142], which protected MN and preserved neuromuscular function. Moreover, Dodge et al. showed that delivering of AAV1- and AAV2-IGF-1 to the CNS of SOD1-G93A mice is sufficient to delay disease progression and demonstrated for the first time that IGF-1 attenuates the pathological activity of non-neuronal cells that contribute to disease progression [143]. Afterwards, the same group evaluated the effect of AAV4-mediated delivery of VEGF or IGF-1 that showed delayed motor decline and significantly extended survival in SOD1-G93A mice [144]. Interestingly, delivery of both neurotrophic factors simultaneously was not more efficacious than the single treatments with either IGF-1 or VEGF. However, combining neurotrophic factor for the treatment of ALS or other MNDs, such as SMA [145], seems a promising perspective. Furthermore, the use of gene therapy tools can directly target the genetic mutation and in parallel act on other important pathways that would protect other disease mechanisms [146,147]. These approaches are currently being explored in further detail.

## 7. Conclusions

Different gene therapy approaches developed for the treatment of ALS are being vigorously pursued. Currently, ASOs for two genetic forms are being tested in clinical trials and the use of viral-mediated gene therapy for ALS is especially promising for a specific and stable expression of the therapeutic agent into the CNS. While LV vectors were used in animal models for proof-of-concept studies, AAV-mediated approaches hold concrete translational perspectives, in particular for fALS cases. Several strategies to treat fALS are being developed and some of them—following the success of the gene therapy strategy for SMA—could enter into clinical trials in the near future. Continual research efforts are required to identify pathological mechanisms for sALS and to develop appropriate therapeutic approaches. With AAV vectors it will be possible to target specific cell types involved in the disease and to deliver different kind of transgenes—to silence, to add therapeutic factors, or to genetically correct. Persistent research efforts in this direction could lead to interventions on common pathways in sALS and fALS but, also, hopefully, for other MNDs. 

## Figures and Tables

**Table 1:** This table summarizes the different approaches for *in vivo* gene transfer.

Tools for In Vivo Gene Therapy and Their Characteristics			
Non-viral strategies	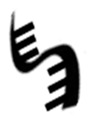 Antisense Oligonucleotides (ASO)	✓ 13–25 nucleotide long, single-stranded nucleic acid (RNA or DNA) ✓ Induction of mRNA degradation through activation of RNaseH ✓ In clinical trial for SOD1-ALS and C9-ALS ✓ ASOs can be produced by plasmids encoding for small nuclear RNA particles (such as modified U7)	ASOs and siRNAs are rapidly degraded by endonucleases and require repeated invasive injection into the central nervous system for ALS treatment. When encoded by plasmids they can be delivered using viral vectors for stable transduction.
	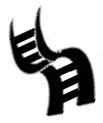 Small Interfering RNAs (siRNA)	✓ 19–23 nucleotide long, double-stranded RNA ✓ Induction of mRNA degradation through nucleases activity ✓ They can be continuously produced by plasmids encoding short-hairpin RNA or artificial microRNA	
Viral vector-mediated strategies	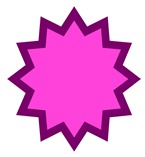 Lentiviral (LV) vectors	✓ Large cloning capacity (8–10 Kb) ✓ Transduction of dividing and non-dividing cells ✓ Long-term transgene expression in dividing cells	LV have a broad tropism and transduce areas close to the injection site. They integrate into the host genome and have a mutagenic risk.
	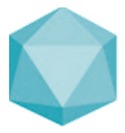 Adeno-Associated virus (AAV) vectors	✓ Transduction of dividing and non-dividing cells ✓ Non pathogenic ✓ Specific tropism for different cell types, according to serotypes ✓ Persistence in the cells as extra-chromosomal episomes ✓ Low risk of insertional mutagenesis	AAV have many advantages for clinical application, but they have a small cloning capacity (single stranded: about 4.7 Kb; self complementary: about 2.4 Kb).

## Abbreviations

AAP	Assembly-Activating Protein
AAV	Adeno-Associated virus
ALS	Amyotrophic Lateral Sclerosis
ASO	Antisense oligonucleotides
B6.SOD1-G93A	SOD1-G93A mouse model backcrossed the to the C57BL/6J
BAC	Bacterial artificial chromosome
BDNF	Brain derived neurotrophic factor
C9-100-1000	BAC C9orf72 mice harboring from 100 to 1000 repeats
C9-450	BAC C9orf72 mice harboring 450 repeats
C9-500	BAC C9orf72 mice harboring 500 repeats
C9orf72	Chromosome 9 open reading frame 72
CHMP	Committee for Medicinal Products for Human Use
CNS	Central nervous system
CRISPR	Clustered regularly interspaced short palindromic repeats
CSF	Cerebrospinal fluid
DPRs	Dipeptide repeat proteins
EIAV	Equine infectious anaemia virus
EMA	European Medicines Agency
fALS	Familial ALS
FDA	Food and Drug Administration
FTD	Frontotemporal dementia
FUS	Fused in sarcoma
G_4_C_2_	GGGGCC
G93A	single amino acid substitution of glycine to alanine at codon 93 of the human SOD1
GA	Glycine–alanine
GDNF	Glial cell line derived neurotrophic factor
GP	Glycine–proline
GR	Glycine–arginine
GWAS	Genome-wide association studies
HiRet	Highly-efficient retrograde gene transfer
HIV	Human immunodeficiency virus
HRE	Hexanucleotide repeat expansion
IGF-1	Insulin-like growth factor 1
iPSC	Induced pluripotent stem cells
ITRs	Inverted terminal repeats
IV	Intravenous
LTR	Long terminal repeats
LV	Lentiviral vectors
miR	microRNA
MN	Motor neurons
MND	Motor neuron disease
MNDs	Motor neuron disorders
NeuRet	Neuron-specific retrograde gene transfer
NMD	Nonsense Mediated Decay
PA	Proline–alanine
PR	Proline–arginine
RAN	Repeat-associated non-AUG-dependent translation
RBPs	RNA-binding proteins
RISC	RNA-induced silencing complex
RNAi	RNA interference
RNase H	Ribonuclease H
RVG	Rabies virus envelope glycoprotein
sALS	Sporadic ALS
Sc	Self-complementary
shRNA	Short hairpin RNA
siRNA	Small interfering RNA
SMA	Spinal Muscular Atrophy
SMN	Survival of motor neuron
SOD1	Cu/Zn superoxide dismutase 1 enzyme
SOD1-G93A	SOD1 mouse model in the mixed B6SJL genetic backgroundexpressing multiple copies of the human SOD1 transgene, carrying a single amino acid substitution of glycine to alanine at codon 93
Ss	Single-stranded
TDP-43	TAR DNA binding protein of 43 kDa
VEGF	Vascular endothelial growth factor
VSV-G	Vesicular stomatitis virus glycoprotein
